# The Genetic and Environmental Foundation of the Simple View of Reading in Chinese

**DOI:** 10.1371/journal.pone.0047872

**Published:** 2012-10-24

**Authors:** Connie Suk-Han Ho, Bonnie Wing-Yin Chow, Simpson Wai-Lap Wong, Mary M. Y. Waye, Dorothy V. M. Bishop

**Affiliations:** 1 Department of Psychology, The University of Hong Kong, Hong Kong, China; 2 Department of Applied Social Studies, City University of Hong Kong, Hong Kong, China; 3 Department of Psychological Studies, Hong Kong Institution of Education, Hong Kong, China; 4 School of Biomedical Sciences, The Chinese University of Hong Kong, Hong Kong, China; 5 Department of Experimental Psychology, University of Oxford, Oxford, United Kingdom; University of Leicester, United Kingdom

## Abstract

The Simple View of Reading (SVR) in Chinese was examined in a genetically sensitive design. A total of 270 pairs of Chinese twins (190 pairs of monozygotic twins and 80 pairs of same-sex dizygotic twins) were tested on Chinese vocabulary and word reading at the mean age 7.8 years and reading comprehension of sentences and passages one year later. Results of behavior-genetic analyses showed that both vocabulary and word reading had significant independent genetic influences on reading comprehension, and the two factors together accounted for most but not all of the genetic influences on reading comprehension. In addition, sentence comprehension had a stronger genetic correlation with word reading while passage comprehension showed a trend of stronger genetic overlap with vocabulary. These findings suggest that the genetic foundation of the SVR in Chinese is largely supported in that language comprehension and decoding are two core skills for reading comprehension in nonalphabetic as well as alphabetic written languages.

## Introduction

The simple view of reading (SVR) comprehension first proposed by Gough and Tunmer [1] states that reading comprehension is the product of only two constructs, listening comprehension and decoding. This view has been extensively explored and generally supported in alphabetic writing systems, primarily English. Behavior-genetic studies with identical and fraternal twins learning to read English have shown that the independent contributions of decoding and listening comprehension to reading comprehension are largely based on independent genetic influences. The present study is unique in its extension of behavior-genetic research on the SVR to a non-alphabetic writtten language, Chinese. We introduce our study by briefly reviewing evidence for the SVR in behavioral studies. Then we review recent behavior-genetic research on the SVR, followed by an overview of the unique characteristics of the Chinese written language and previous research on reading comprehension in Chinese.

### The Simple View of Reading Comprehension

The SVR has gained support from regression and structural equation modeling analyses showing that most if not all of the reliable individual differences in reading comprehension can be accounted for by word decoding and listening comprehension, and each component makes significant unique contributions to reading comprehension [2], [3]. In other words, decoding and listening comprehension are the core skills that are necessary for reading comprehension. The SVR is also a useful tool to classify children’s reading impairments. Dyslexic children are found mainly to have decoding problems while poor comprehenders have difficulties in language comprehension. Generally poor readers have difficulties in both [4], [5]. The existence of poor word decoders and poor comprehenders indicates some independence between decoding and comprehension skills [6], [7].

Despite the beauty and simplicity of the model, its components have not been clearly defined and clear definition of the constructs is important for validation of the model. For instance, ‘LC’ in the SVR may refer to oral language comprehension, which includes a range of verbal language skills like vocabulary, syntax, inferencing, and the construction of mental schemas. We prefer to use the term “language comprehension” for “LC”, so as not to confuse with the listening comprehension measures used in many studies. ‘D’ may mean successful word reading or the ability to use phonological decoding (as normally measured by nonword reading). Kirby and Savage suggested that ‘D’ should be defined as word recognition [8]. However, word recognition involves a range of skills beyond phonological decoding, including orthographic processing, and rapid naming. As for ‘RC’, there are different levels of text understanding (e.g., literal vs. inferential) and the length and style of the text may vary. It has been found that different RC measures may involve different cognitive constructs [9], [10]. This point will be discussed in greater details in later sections.

### Behavioral Genetic Studies Examining the Simple View of Reading

The SVR has gained considerable recognition in the field, with validation in many behavioral studies. It is interesting to know whether the SVR has a genetic foundation as well. In other words, do language comprehension and word decoding account for the genetic influence on reading comprehension, and do they make independent genetic contributions to reading comprehension? Several behavior-genetic studies have examined this issue. Keenan and her colleagues were the first to test the SVR in a behavior-genetic twin design [11]. They tested 70 pairs of English-speaking identical twins and 121 pairs of fraternal twins of 8 to 17 years of age in Colorado. They employed three listening comprehension tasks as measures of the language comprehension construct, two word recognition tasks with or without time constraint for measuring the decoding construct, and four reading comprehension measures that covered a range of discourse of different test formats. Their major findings were (a) there were significant independent genetic contributions of listening comprehension and word decoding to reading comprehension, and (b) listening comprehension and word decoding together accounted for all the genetic influence on reading comprehension. Harlaar et al. extended Keenan et al.’s work by including measures of phonological decoding and word recognition to index word decoding, and measures of vocabulary and listening comprehension to assess oral language skills [12]. Their results replicated those of Keenan et al. in that word decoding and oral language skills accounted for all the genetic influences on reading comprehension and there were etiological links (both genetic and environmental) between oral language and reading comprehension that were largely independent of word decoding. These findings have provided solid evidence that supports the SVR in genetic terms. Since Keenan et al.’s study examined children of a wide age range at one time point, it would be interesting to know whether the same pattern of results also applies to longitudinal examination of children over a smaller age range.

Byrne et al. reported the results from 167 pairs of identical twins and 152 pairs of fraternal twins at Grade 1 of their International Longitudinal Twin Study (ILTS) [13]. They found that the genetic correlation between word reading and reading comprehension was very strong (.97). No result was reported regarding the genetic correlation between reading comprehension and listening comprehension measures, but the genetic correlation between word recognition and reading comprehension was so high that there could be no independent genetic influence from listening comprehension. With inclusion of more participants in Grade 2 (303 identical twin pairs and 312 fraternal twin pairs), Byrne et al. later reported a similarly strong genetic correlation between word reading and reading comprehension (.88) [14]. However, the genetic correlation between vocabulary and reading comprehension was much lower (.46). These results suggest that a single genetic factor is associated with decoding and reading comprehension in the early grades.

Olson et al. followed up to report the results of their children in ILTS up to the end of Grade 4 [15]. In contrast to Byrne et al.’s findings [14] they found that the genetic correlation between vocabulary and reading comprehension approached unity (.97) by Grade 4. Again, vocabulary and word recognition accounted for all of the genetic influences on reading comprehension. Taking the findings of Byrne et al. and Olson et al. together [13], [14], [15], it appears that reading comprehension is genetically more associated with word decoding for children at the beginning stage of learning to read (Grades 1 and 2) but later it is more strongly associated with language comprehension (as measured by vocabulary). This is in line with findings in other behavioral studies. At the beginning stage of learning to read, children are learning to decode and identify words, and this word-reading process limits their comprehension. This is why the correlations between reading comprehension and decoding are much stronger than that with language comprehension at this stage both phenotypically [16], [17], [18] and genetically [15], [19]. When children’s word decoding skills become automatic later, their reading comprehension depends mainly on their language comprehension skills as seen by stronger correlations between the two both phenotypically [20] and genetically [15].

In addition, several longitudinal twin studies have also confirmed strong longitudinal genetic and environmental correlations between early language skills (e.g., vocabulary and syntax) and later reading performance [15], [21], [22]. These findings have demonstrated the long reach of genetic and environmental influences on preschool oral language skills to later reading development. These authors have suggested that some shared family and school environments that are good for promoting oral language skills may in turn facilitate reading development.

However, it was noted that the reading comprehension measure in Grade 2 of Olson et al.’s study [15], the Woodcock Passage Comprehension test, employed short passages of one to two sentences and the child was asked to orally provide a single missing word. Their Grade 4 measure, the Gates-MacGinitie test of reading comprehension, included a series of longer passages of four to six sentences that the child was asked to answer multiple-choice questions. Apart from the age difference, the differences in length and test format of the reading comprehension measures may also be responsible for the different patterns of correlations in the two grades.

Keenan, Betjemann and their colleagues have examined the effect of different reading comprehension measures on the outcomes of genetic analyses [9], [19], [23]. They found that two tests (Woodcock-Johnson Passage Comprehension and Peabody Individual Achievement Test) were most strongly associated with decoding (which were called RC-D measures), and three other tests (Gray Oral Reading Test, Qualitative Reading Inventory Questions and Retell) were more associated with listening comprehension (which were called RC-LC measures), both phenotypically and genetically. The RC-D measures required silent reading of short passages of one to two sentences. The child was asked to provide orally a missing word in one measure and to select from four pictures one best represented the meaning of the sentence in another measure. The RC-LC measures included longer passages up to 785 words. Test format included multiple-choice comprehension questions, open-ended short-answer questions, and retelling the passage. They suggested that passage length was one of the key factors why the different reading comprehension measures loaded differently on decoding vs. listening comprehension. For reading single sentences, normally successful decoding of core words determines good understanding of the sentence. When the passages are long, decoding problems may be rectified with the help of context. Therefore, different reading comprehension measures may assess very different cognitive and literacy skills.

So far all of the reported studies examining the SVR, either phenotypically or genetically, have been conducted in English. It would theoretically be interesting to examine the genetic foundation of the SVR in a non-alphabetic language to examine the universality of the model. Chinese is a good test case given its distinct linguistic features. Since readers may not be familiar with these, we will first describe briefly the characteristics of the Chinese language.

### Characteristics of the Chinese Language

The basic graphic unit in Chinese is a character. The fact that the Chinese character is simultaneously a visual whole, a syllabic unit, and a morpheme contrasts with the units of writing in alphabetic scripts, letters, which indicate sound only and have no dovetailed relation with meaning. The script-sound-meaning convergence of the Chinese character may facilitate the process of understanding and retrieval of the meaning of multicharacter words [24]. One obvious advantage of this logographic and morphosyllabic nature of the Chinese language is that the same script can be used in a large population where people speak different dialects.

About 80% to 90% of Chinese characters are ideophonetic compounds, each comprising a semantic and a phonetic component (stroke-pattern known as radical). In general, the semantic radical in a Chinese character signifies the semantic category of the character, while the phonetic radical provides sound cues of a Chinese character directly from its own pronunciation or indirectly by making an analogy with other characters having the same phonetic radical. Overall, semantic radicals are functionally more reliable than phonetic ones. As such, Chinese characters provide more meaning than sound cues and may facilitate the learning of word meaning and text comprehension.

### Chinese Twin Study of Reading Development

Given the special characteristics of the Chinese language, the Chinese Twin Study (CTS) of reading development was the first to investigate genetic and environmental influences on Chinese language and reading abilities and to examine whether the roles of heredity and environment would be similar to those in alphabetic languages. Chow and her colleagues reported that the genetic contributions to word reading, phonological memory and rapid naming, and the shared environmental influences on receptive vocabulary are likely to be universal across languages; whereas the importance of shared environment on rhyme and syllable awareness seems to be unique to Chinese [25]. The present study was part of the CTS and extended Chow et al.’s examination from word level processing to reading comprehension in Chinese.

### Research Findings Related to Reading Comprehension in Chinese

Although the SVR has not been examined in Chinese, findings of some studies do give us insights regarding the validity of the model in Chinese. For instance, a study by Shu and her colleagues found that vocabulary significantly predicted Chinese reading comprehension after adjusting for morphological awareness, rapid naming and phonological awareness among Mandarin-speaking children in Beijing [26]. However, vocabulary did not significantly predict reading comprehension in a study with Grade 1 children in Hong Kong [27]. In the study by Chik et al., oral vocabulary, which was assessed by children’s ability to verbalize their knowledge of a word’s meaning (i.e., a measure of vocabulary depth), predicted reading comprehension among Grades 1 to 3 children, but not among Grade 4 to 5 children in Hong Kong [28].

It is noteworthy that in Hong Kong, the Chinese dialect spoken by the majority of Chinese people is Cantonese. While there is high consistency between the written form of Chinese and the dialect of Mandarin, Cantonese differs in some ways from Modern Standardized written Chinese in both vocabulary and syntax. We might therefore expect that the link between oral vocabulary and reading comprehension in Cantonese-speaking children will be less prominent compared to those found among Mandarin-speaking Chinese and English-speaking children.

The role of listening comprehension was also examined in Yeung et al.’s study [27]. However, they did not find a significant link between listening comprehension and reading comprehension in their Cantonese-speaking first graders. Again one possibility was related to a weaker linkage of oral language and literacy skills in their Cantonese-speaking participants. Another reason may be that the reliability of their listening comprehension task was less than satisfactory (.38).

In the same study, Yeung et al. also reported that word reading has a stronger association with sentence comprehension (with a path coefficient of.64) than with passage comprehension (with a path coefficient of.32) in their model [26]. They also found different significant contributors to sentence and passage comprehension in Grade 1. Specifically, orthographic skills (as measured by knowledge of semantic radicals) and syntactic skills were found to contribute significantly to sentence comprehension, while rapid naming and syntactic skills were significant predictors of passage comprehension after controlling for the effects of word reading and other variables. In addition, they reported the significant role of discourse skills in passage comprehension for the same group of children in Grade 4 [29]. Therefore, reading sentences and passages do require a different set of skills. Reading sentences relies more on word decoding, orthographic skills like semantic radical knowledge, and syntactic skills. Knowledge of the semantic category of semantic radicals may contribute to the understanding of word meaning, which in turn facilitates sentence understanding. Understanding syntactic sentence structure also facilitates understanding the relationships among components in a sentence. On the other hand, reading passages requires automatic and rapid retrieval of information that frees resources for higher level processing. In addition to syntactic skills, discourse skills help the reader to develop the mental schema of the passage.

### Aims of the Present Study

The present study aimed to test the SVR in Chinese from a behavioral genetic perspective. The specific research questions were: (1) whether word decoding and oral language comprehension have significant independent genetic influences on reading comprehension in Chinese, (2) whether word decoding and language comprehension accounted for most if not all the genetic influences on reading comprehension in Chinese, and (3) whether word decoding and language comprehension contributed differently in genetic terms to written sentence comprehension and passage comprehension in Chinese. These questions were addressed with a group of Chinese twins recruited in Hong Kong, China. The genetic and environmental associations of word decoding and language comprehension with reading comprehension were examined. Results are compared with findings in the English studies to examine the universality of the SVR. According to Hoover and Gough, decoding could be assessed by the ability to pronounce isolated real words and pseudowords [30]. For beginning readers, meaning in the mental lexicon could be accessed with phonological codes already developed through the course of language acquisition [30]. Since there is no phonological decoding per se in Chinese, word reading was used to index decoding. We believe that oral vocabulary is a core aspect of language comprehension and Olson et al. have demonstrated that the genetic correlation between vocabulary and reading comprehension approached unity (.97) among Grade 4 twins [15]. It appears that vocabulary captures a large proportion of oral language comprehension that overlaps with reading comprehension, especially for older readers. Oral vocabulary was thus used as a proxy of language comprehension, and there were separate measures for two aspects of reading comprehension, sentence and passage comprehension.

## Methods

### Ethics Statement

Ethical approval was obtained from the Human Research Ethics Committee for Non-clinical Faculties of the University of Hong Kong. Parental written consent was obtained for each participant.

### Participants

The present paper reports data from the Chinese Twin Study (CTS) of reading development. The CTS included a sample of 388 pairs of unselected monozygotic (MZ) and same-sex dizygotic (DZ) twins from Hong Kong, China. All the kindergartens and elementary schools in Hong Kong were invited to participate. Written consent was sought from the parents of participating twins. The same-sex DZ to MZ twin ratio was 0.35 in the whole CTS sample. The DZ and MZ twinning ratio tends to be lower in Asian populations [31]. With opposite-sex twin pairs included, the DZ to MZ twin ratio was approximately 0.65 for twins born to Chinese fathers or mothers [32]. Thus, the same-sex DZ to MZ twin ratio should be around 0.33 with the assumption of equal number of same-sex and opposite-sex DZ twin pairs. Therefore, the proportion of twin types in the CTS sample was comparable to that of the population prevalence. Children in the CTS sample had been tested once annually for three years on a broad range of speech, language, cognitive, and literacy skills.

In the present study, we focused on the CTS measures of oral vocabulary and word reading administered in the second year, and measures of reading comprehension in the third year of the study. Children who participated in all the assessments and were at age 5 or above were selected for the present analyses. In general, children in Hong Kong enter kindergarten and primary school at around age 3 and age 6 respectively, and are provided with 3 years of kindergarten education and 6 years of primary education. Children in Hong Kong are typically introduced to reading and writing of Chinese at age 4 years or below. Thus all children had received reading instruction for at least one year. All the children were given an audiometric screening test to ensure they had normal-range hearing for speech frequencies. Three children, who could not hear 35 dB or above with the better ear, were excluded. This final selected sample consisted of 190 pairs of MZ twins (96 male pairs and 94 female pairs) and 80 pairs of DZ twins (48 male pairs and 32 female pairs) aged from 5 to 11.5 years with a mean age of 7.8 years and a SD of 1.6 years when they were first assessed on these measures. SNP testing was conducted to determine twin pairs’ zygosity.

### Measures

The measures included in the present study were from the larger test batteries that were administered in the CTS and some of the findings of the first year of the study had been reported [25].

#### Nonverbal reasoning

The 36-item Raven’s Coloured Progressive Matrices (RCPM) [33] was employed to assess children’s nonverbal reasoning. As this task has not yet been normed on the Chinese population, raw scores were used. The maximum score of this test was 34 (excluding two trial items) and the Cronbach’s alpha was .93.

#### Receptive vocabulary

The receptive vocabulary test consisted of 2 practice and 80 test items translated and adapted for Chinese from the Peabody Picture Vocabulary Test – Fourth Edition (PPVT-IV) [34]. For each item, the experimenter read out the target item and the child was required to select a picture from the four options to match it. These items were ranked in increasing difficulty. Correct responses given in 9 or all items in the first 10 consecutive items fulfilled the basal rule. Testing stopped when the child failed to identify 15 consecutive items. The maximum score of this test was 80 and the Cronbach’s alpha was .93.

#### Word reading

The word reading test consisted of a total of 198 items, namely 48 items of single-character Chinese words and 150 items of two-character Chinese words. The single-character words were taken from popular Chinese language textbooks of kindergarten levels in Hong Kong based on the results of a pilot study. These words were included particularly for the younger children in the present study. The 150 two-character words were taken from the Chinese Word Reading subtest of the Hong Kong Test of Specific Learning Difficulties in Reading and Writing (HKT-SpLD) [35]. The HKT-SpLD is a standardized test developed for Hong Kong primary school children, and items in the Chinese Word Reading subtest are common two-character words of Grade 1 to Grade 6 levels. The words were arranged in an order of increasing difficulty. The child was required to read each word aloud. Testing stopped when the child failed to read 15 consecutive items. The maximum score was 198 and the Cronbach’s alpha was .99.

#### Reading comprehension 1: Sentences

Children’s reading comprehension of sentences was assessed by a cloze sentence task. There were 16 written cloze sentences with a noun, a verb, or an adjective missing in each sentence. Some of the sentences were adapted from Yeung et al’s and Chik et al’s studies [27], [28]. The sentences were of Grade 1 to Grade 5 levels. Words and sentence types of these sentences were taken from popular Chinese language textbooks and exercise books of elementary levels in Hong Kong. The sentences were printed on pieces of A4 paper. The participants were asked to read each sentence carefully and to choose, from four choices, the word that best completed the sentence. All four choices in the same item were of the same word class and taken from word pools of a similar grade level but were different in terms of meaning and usage. To arrive at the correct answer, the child needs to make use of and integrate the information available in each cloze sentence. The participants were given two practice items with corrective feedback before the testing ones. There was no time limit and the children completed the task at their own pace. The maximum score was 16, and its Cronbach’s alpha was .89.

**Table 1:** Mean Raw Scores (and Standard Deviations) of the Measures and the Intra-class Twin Correlations for standardized residuals (controlling for age).

Measure (Max.)	Mean (SD)	Correlation	
		MZ	DZ
Vocabulary (80)	61.82 (11.78)	.69	.45
Word reading (198)	114.64 (52.61)	.92	.55
Sentence comprehension (16)	11.08 (4.26)	.64	.42
Passage comprehension (12)	6.31 (2.90)	.53	.25

Note. All correlation coefficients were significant at p<.001 except that of passage comprehension for DZ (p<.05).

**Table 2:** Test Intercorrelations among All the Measures (standardized residuals).

Measure	Vocabulary	Word reading	Sentence comprehension	Passage comprehension
**Vocabulary**		.32	.33	.39
**Word reading**			.68	.44
**Sentence comprehension**				.38

Note. All correlation coefficients were significant at p<.001.

#### Reading comprehension 2: Passages

This reading comprehension task consisted of two narrative passages and one expository passage. Some of the passages and questions were adapted from Yeung et al’s and Chik et al’s studies [27], [28]. Narrative and expository passages are the two most common types of genre found in the Chinese language textbook of elementary school students in Hong Kong. There were four types of questions, with the first one developed by the authors and the last three based on the question types for reading comprehension in the Progress in International Reading Literacy Study (PIRLS) 2006: (1) explain vocabularies from the text in which its meaning could be derived from understanding the passage, (2) focus on and retrieve explicitly stated information and ideas, (3) make straightforward inferences, and (4) interpret and integrate ideas and information. The passages were of Grade 1 to Grade 5 levels. The length of passages ranged from 104 to 160 characters. There were 4 multiple-choice questions in each passage and each question had four answer choices. Contents of same-item answer choices contained overlapping information (e.g., all related to Autumn, part of the correct answer) or information of the same nature (e.g., all were numbers) and their lengths were similar in most questions. All the passages and questions were printed on pieces of A4 paper and presented visually to the participants. A short passage with two questions was given to the children for practice (with corrective feedback) before they read the test passages. There was no time limit and the children completed the task at their own pace. The maximum score for this task was 12, and its Cronbach’s alpha was .72.

**Table 3:** Estimates of genetic (a^2^), shared environmental (c^2^), and unique environmental (e^2^) contributions to the variance within each measure.

Variable	a^2^	c^2^	e^2^
Vocabulary	0.50 (0.20, 0.75)	0.19 (0.00, 0.47)	0.31 (0.24, 0.38)
Word reading	0.75 (0.52, 0.93)	0.16 (0.00, 0.40)	0.08 (0.06, 0.11)
Sentence comprehension	0.51 (0.18, 0.71)	0.14 (0.00, 0.44)	0.36 (0.28, 0.44)
Passage comprehension	0.53 (0.17, 0.62)	0.00 (0.00, 0.32)	0.47 (0.38, 0.57)

Note. 95% confidence intervals are in parentheses.

### Procedure

Each child was tested individually for around one hour each time on a battery of tests by trained research assistants, psychology major undergraduates or graduates in their school, their home, or our laboratory in Hong Kong according to the parents’ preference. Saliva was collected from co-twins with DNA kits for zygosity assessment.

## Results

To adjust for age effects, the raw scores of each task were regressed on children’s age. It was found that a quadratic function fitted most of the measures better than a linear function did. Therefore, quadratic regression was applied on all the measures and the standardized residual scores regressing on age were used in all later analyses.

**Figure 1:**
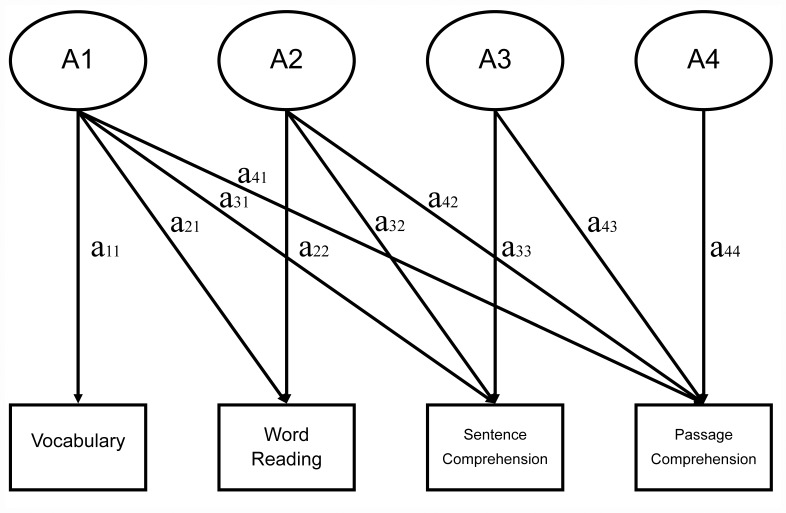
Genetic component of the Cholesky decomposition model for oral vocabulary, word reading, sentence comprehension, and passage comprehension.

**Table 4:** Genetic and Environmental Influences on Vocabulary, Word Reading, Sentence Comprehension, and Passage Comprehension.

	A1	A2	A3	A4
**Vocabulary**	0.65*** (0.43, 0.88)			
**Word reading**	0.25 (−0.04, 0.54)	0.83*** (0.67, 0.98)		
**Sentence comp.**	0.14 (−0.15, 0.43)	0.51*** (0.32, 0.71)	0.32*** (0.16, 0.49)	
**Passage comp.**	0.42** (0.12, 0.73)	0.25* (0.03, 0.48)	0.46*** (0.28, 0.63)	0.00 (−1.20, 1.20)
	**C1**	**C2**	**C3**	**C4**
**Vocabulary**	0.51*** (0.23, 0.79)			
**Word reading**	0.21 (−0.16, 0.59)	0.36 (0.00, 0.72)		
**Sentence comp.**	0.43* (0.09, 0.78)	0.21 (−0.31, 0.72)	0.12 (−0.38, 0.63)	
**Passage comp.**	0.09 (−0.32, 0.51)	0.21 (−0.25, 0.68)	−0.15 (−0.75, 0.44)	0.00 (−0.50, 0.50)
	**E1**	**E2**	**E3**	**E4**
**Vocabulary**	0.56*** (0.50, 0.61)			
**Word reading**	0.05** (0.01, 0.09)	0.28*** (0.25, 0.31)		
**Sentence comp.**	0.00 (−0.08, 0.08)	0.26*** (0.17, 0.34)	0.55*** (0.50, 0.60)	
**Passage comp.**	0.01 (−0.08, 0.11)	0.17*** (0.08, 0.27)	−0.08 (−0.17, 0.00)	0.66*** (0.60, 0.72)

Note. 95% confidence intervals are in parentheses.

* p<.05,

** p<.01,

*** p<.001.

### Phenotypic Analyses

Table 1 shows the mean raw scores, standard deviations, and intra-class twin correlations of the measures. Twin correlations were computed using the standardized residual scores. Table 2 shows the intercorrelations among the measures using the standardized residual scores of a randomly selected cotwin from each twin pair. It was found that correlation among all the measures was significant (all *r*s >.31, all *p*s <.001). In general, reading comprehension correlated more strongly with word reading (r = .68 and.44) than with vocabulary (r = .33 and.39).

**Table 5:** Genetic and Environmental Influences on IQ, Vocabulary, Word Reading, Sentence Comprehension, and Passage Comprehension.

	A1	A2	A3	A4	A5
**IQ**	0.78*** (0.61, 0.95)				
**Vocabulary**	0.33** (0.08, 0.58)	0.56*** (0.34, 0.79)			
**Word reading**	0.30* (0.05, 0.54)	0.12 (−0.19, 0.44)	0.80*** (0.64, 0.95)		
**Sentence comprehension**	0.34** (0.10, 0.59)	−0.02 (−0.34, 0.31)	0.44*** (0.22, 0.66)	0.27* (0.05, 0.48)	
**Passage comprehension**	0.42*** (0.19, 0.64)	0.22 (−0.05, 0.49)	0.22* (0.02, 0.43)	0.42*** (0.26, 0.57)	0.00 (−1.08, 1.08)
	**C1**	**C2**	**C3**	**C4**	**C5**
**IQ**	0.24 (−0.24, 0.72)				
**Vocabulary**	0.12 (−0.62, 0.87)	0.50*** (0.26, 0.74)			
**Word reading**	0.00 (−0.80, 0.80)	0.21 (−0.14, 0.56)	0.37* (0.01, 0.73)		
**Sentence comprehension**	−0.09 (−0.83, 0.66)	0.44** (0.13, 0.76)	0.23 (−0.27, 0.73)	0.00 (−0.52, 0.52)	
**Passage comprehension**	0.21 (−0.37, 0.78)	0.07 (−0.24, 0.37)	0.19 (−0.20, 0.58)	0.00 (−0.56, 0.56)	0.00 (−0.42, 0.42)
	**E1**	**E2**	**E3**	**E4**	**E5**
**IQ**	0.58*** (0.52, 0.64)				
**Vocabulary**	0.03 (−0.05, 0.11)	0.56*** (0.50, 0.61)			
**Word reading**	0.03 (−0.01, 0.07)	0.05* (0.01, 0.09)	0.28*** (0.25, 0.31)		
**Sentence comprehension**	0.00 (−0.08, 0.09)	0.00 (−0.09, 0.08)	0.26*** (0.18, 0.34)	0.54*** (0.49, 0.59)	
**Passage comprehension**	0.06 (−0.03, 0.15)	0.01 (−0.08, 0.10)	0.16*** (0.07, 0.26)	−0.08 (−0.17, 0.01)	0.66*** (0.59, 0.72)

Note. 95% confidence intervals are in parentheses.

* p<.05,

** p<.01,

*** p<.001.

### Genetic Analyses

To address the several research questions in the present study, univariate and multivariate genetic analyses were conducted with OpenMx in the R statistical modeling package [36]. Table 3 shows the proportions of variance in each measure due to genetic (a^2^), shared environmental (c^2^) (i.e., external factors which contribute towards the resemblance among individuals growing up in the same environment, e.g., being taught by the same teacher), and unique environmental effects (e^2^) (i.e., individual specific factors that create differences among co-twins from the same family, e.g., an accident). Overall, word reading had strong heritability (a^2^ = .75), while vocabulary and reading comprehension had moderate genetic influence (a^2^ ranged from .50 to .53). The environmental influences common to both twins were far weaker than those of the genetic ones (c*^2^* ranged from 0 to.19) and the unique environmental influences were moderate (e*^2^* ranged from .08 to .47).

**Table 6:** Genetic and Environment Correlations among All the Measures.

	Genetic		
	1.	2.	3.
**1. Vocabulary**	–		
**2. Word reading**	0.29	–	
**3. Sentence comp.**	0.22	0.86	–
**4. Passage comp.**	0.63	0.54	0.80
	**Shared Environment**		
	**1.**	**2.**	**3.**
**1. Vocabulary**	–		
**2. Word reading**	0.51	–	
**3. Sentence comp.**	0.88	0.80	–
**4. Passage comp.**	0.34	0.83	0.48
	**Unique Environment**		
	**1.**	**2.**	**3.**
**1. Vocabulary**	–		
**2. Word reading**	0.19	–	
**3. Sentence comp.**	0.00	0.42	–
**4. Passage comp.**	0.02	0.25	0.00

Multivariate genetic analyses using the Cholesky decomposition model were also conducted to investigate the genetic and environmental links among vocabulary, word reading, and reading comprehension in Chinese. Cholesky decomposition breaks down phenotypic covariances among variables into their shared and independent variance associated with genes, shared environment, and unique environment. Figure 1 shows the additive genetic paths (*a11 to a44*) from factors A1 (vocabulary), A2 (word reading), A3 (sentence comprehension), and A4 (passage comprehension). The full model also includes corresponding factors and paths for shared environment (*C, c)* and unique environment (*E, e*). Table 4 presents the Cholesky results of the genetic (A), shared environmental (C), and unique environmental (E) standardized path estimates of the four reading-related measures. If we looked at the specific path estimates, we found that vocabulary (A1) shared significant genetic influence with passage comprehension (.42). Word reading (A2) also shared significant genetic influence with both sentence comprehension (.51) and passage comprehension (.25) after controlling for the genetic influences on vocabulary. Sentence comprehension (A3) also shared significant genetic influence with passage comprehension (.46) after controlling for vocabulary and word reading. Finally, there was no genetic influence left on passage comprehension (A4) after controlling for the first three factors.

The environmental influences common to both twins were far weaker than those of the genetic ones. The common environmental influences on vocabulary were shared significantly only with sentence comprehension (.43). As for the unique environmental influences, all of the large significant effects were on the diagonal, which were specific to each factor, and might be a result of test measurement error (e.g., an experimenter giving prompts to a participant more than needed in a test).

Next, we would like to see how these reading-related variables relate to each other after controlling for IQ. IQ was entered first in the multivariate genetic analyses and followed by vocabulary, word reading, sentence comprehension, and passage comprehension. Table 5 shows that IQ was highly heritable in this sample (a^2^ = .61) and it had significant genetic paths to all the other variables. The result patterns with or without IQ controlled were very similar. The only exception was that vocabulary no longer shared significant genetic influence with passage comprehension when IQ was controlled.

Based on the above results, vocabulary and word reading together did not account for all the genetic influences on sentence and passage comprehension. There was still significant genetic influence on sentence comprehension (independent a^2^ = .10 when IQ was not controlled) after controlling for vocabulary and word reading. To further examine this, we did an additional analysis by replacing the separate scores of Sentence comprehension and Passage comprehension with a composite reading comprehension score. This composite score was computed by taking the average of the standardized score of Sentence comprehension and that of Passage comprehension. A similar result pattern was found that vocabulary (A1) shared significant genetic influence with reading comprehension (.33). Word reading (A2) also shared significant genetic influence with reading comprehension (.46) after controlling for vocabulary. There was still a significant genetic influence on reading comprehension under A3 (independent a^2^ = .21) after controlling for vocabulary and word reading. To save space, other results were not reported here.

Table 6 shows the genetic and environmental correlations of the first set of multivariate genetic analyses (with separate scores for Sentence and Passage comprehension), which refer to the degree to which phenotypic correlations are due to genetic and environmental influences common to a pair of correlated variables. Vocabulary had a stronger genetic correlation with passage comprehension (.63) than with sentence comprehension (.22). The reverse was true for word reading (*r* = .86 for sentence comprehension, and .54 for passage comprehension). The strongest shared environmental correlations were between vocabulary and sentence comprehension, and between word reading and reading comprehension.

## Discussion

To recap, the primary aim of the present study was to examine the Simple View of Reading (SVR) in Chinese from a behavioral genetic perspective. Results of the multivariate genetic analyses with the Cholesky decomposition show that both vocabulary and word reading have significant shared and independent genetic influences on reading comprehension, like that in English. Vocabulary and word reading together account for most but not all the genetic influences on reading comprehension. Results of the genetic correlations show that sentence comprehension in Chinese has a stronger genetic overlap with word reading than with vocabulary. On the other hand, passage comprehension shows a trend of stronger genetic overlap with vocabulary than with word reading. Although in a sample of this size, some of the genetic correlations do not differ significantly, it is noteworthy that the pattern is similar to that seen for English. Implications of these findings will be discussed in details below.

### The Simple View of Reading in Chinese

To confirm the genetic foundation of the SVR in Chinese, we expect to find that (1) language comprehension and decoding are two core components in reading that they have significant independent genetic influences on reading comprehension, and (2) language comprehension and decoding together account for all (or most) the genetic influences on reading comprehension. The present findings largely confirm these expectations. In both genetic analyses using the separate scores or composite score of reading comprehension, it was found that vocabulary shared significant genetic influence with reading comprehension, and word reading also shared significant independent genetic influence with reading comprehension when vocabulary was controlled. In other words, there is evidence for both significant shared and independent genetic influences on reading comprehension from both vocabulary and word reading in Chinese. However, in the genetic analyses using separate scores for sentence comprehension and passage comprehension, vocabulary shared significant genetic influence with passage comprehension only (when IQ was not controlled), while word reading shared significant genetic influence with both sentence comprehension and passage comprehension when vocabulary was controlled. In addition, both analyses show that vocabulary and word reading account for a substantial amount of genetic influences on reading comprehension in Chinese.

Unlike findings in some English studies, vocabulary and word reading in the present study together do not account for all the genetic influences on reading comprehension. One possibility is that vocabulary alone does not fully tap the construct of language comprehension in the SVR especially when comprehension of longer passages is examined. There are other linguistic skills, apart from vocabulary, which are essential for explaining individual differences in reading comprehension. For instance, Keenan et al. employed three listening comprehension measures that had tapped a wider range of language skills such as syntactic, semantic, and discourse skills, and these skills are important for understanding longer passages [11]. Harlaar et al. also employed measures of vocabulary and listening comprehension for the construct of language comprehension [12]. Both studies found no residual genetic influences on reading comprehension after controlling for decoding and language comprehension.

### Reading Sentences vs. Reading Passages

There have been several studies in Chinese showing that reading sentences may require a different set of skills as compared with reading passages [27], [29]. Specifically, Yeung and her colleagues have reported that word reading, orthographic skills, and syntactic skills are important for reading sentences, while rapid naming, syntactic skills, and discourse skills are significant unique predictors of passage comprehension. The present findings also suggest different genetic overlap of sentence and passage comprehension with other reading and language skills. Specifically, our findings show that vocabulary has a stronger genetic correlation with passage comprehension than with sentence comprehension in Chinese. The opposite pattern was seen for word reading. This is in line with the findings of Keenan, Betjemann and their colleagues that their RC-D measures were most strongly associated genetically with decoding and the RC-LC measures were more associated with listening comprehension [9], [19], [23]. The RC-D measures required reading of short passages of one to two sentences, which were very much like our Sentence comprehension measure. The RC-LC measures included reading of longer passages like our Passage comprehension task. For reading single sentences, successful decoding of core words is highly important for understanding of a sentence. This may especially be the case for Chinese as Chinese words provide strong meaning cues, which facilitate sentence comprehension. When the passages are long, decoding problems may be rectified with the help of context. Linguistic skills like vocabulary, syntactic, and discourse skills are helpful for understanding the context and structure of longer passages.

Although genetic correlations indicated strongest links between sentence comprehension and word reading, and passage comprehension and vocabulary, the shared environmental correlations indicated that vocabulary, word reading, and reading comprehension share similar environmental influences. This would be consistent with the idea that instructional and home factors that facilitate the development of vocabulary skills should also work for word reading and reading comprehension.

### Comparison with English Findings

To further examine the universality of the SVR, we considered whether the present findings are comparable with those in English. The study that had the best available match of factors and children’s age with the present one was the Grade 2 results reported by Olson et al, whose study was a representative one examining the SVR in English [15]. Their children were around 8.3 years in Grade 2 and the mean age of our sample was 7.8 years. Olson et al. included measures of vocabulary, word recognition, decoding, and reading comprehension. It was noted in their Table 4 that both vocabulary and word recognition had significant unique genetic contribution to reading comprehension. There was still a significant genetic influence on reading comprehension in Grade 2 after controlling for vocabulary, word recognition, and decoding. However, vocabulary and word recognition together accounted for all the genetic influences on reading comprehension in Grade 4. In their Table 5, the genetic correlation of vocabulary with word recognition was .34 (it was .29 in the present study), vocabulary with reading comprehension was .60 (the correlations with sentence comprehension and passage comprehension were .22 and .63 respectively in the present study), and word recognition with reading comprehension was .82 (they were .86 with sentence comprehension and .54 with passage comprehension in the present study). In general, the pattern of results in Chinese was quite similar to that of Olson et al. in English [15]. These findings suggest that the SVR may be universal across languages both phenotypically and genetically.

### Limitations

There are three major limitations in the present study. First, the present sample size was relatively small and there was a wide age range for the participants. A large sample with a smaller age range is desirable for examination of specific developmental patterns longitudinally. Second, because of limitations on testing time, we had only one measure for each construct. It would be ideal to have multiple measures for each construct. This is especially the case for the construct of language comprehension, where having different measures of vocabulary, syntax, and discourse skills would tap the construct more comprehensively. There could also be measures of both accuracy and fluency of reading in future studies. Third, the reading comprehension measures were administered a year later than the word reading and vocabulary measures. Results may be slightly different if all the measures were administered in the same year.

### Conclusions and Future Directions

The present findings have provided some evidence to support the genetic foundation of the SVR in Chinese, like that in English. In other words, language comprehension and word decoding are two core skills that are necessary for reading comprehension in alphabetic and nonalphabetic languages. In particular, decoding skills are more important for reading sentences while language comprehension is more crucial for understanding longer passages. Therefore, when considering reading ability in Chinese children, it is important to recognise that their oral language skills will have a significant impact on their functional literacy.

## References

[1] Gough PB, Tunmer WE (1986) Decoding, reading, and reading disability. REM SPEC EDUC 7(1): 6–10.

[2] Aaron PG, Joshi M, Williams KA (1999) Not all reading disabilities are alike. J LEARN DISABIL-US 32(2): 120–137. 10.1177/002221949903200203 15499713

[3] Catts HW, Hogan TP, Adlof SM (2005) Developmental changes in reading and reading disabilities. In: Catts H, Kamhi A, editors. The connections between language and reading disabilities. Mahwah: Lawrence Erlbaum Associates.

[4] Catts HW, Hogan TP, Fey ME (2003) Sub-grouping poor readers on the basis of individual differences in reading-related abilities. J LEARN DISABIL-US 36(2): 151–164. 10.1177/002221940303600208 PMC2848965 15493430

[5] Nation K, Norbury CF (2005) Why reading comprehension fails: Insights from developmental disorders. TOP LANG DISORD 25(1): 21–32.

[6] Leach JM, Scarborough HS, Rescorla L (2003) Late-emerging reading disabilities. J EDUC PSYCHOL 95(2): 211–224.

[7] Oakhill JV, Cain K, Bryant PE (2003) The dissociation of word reading and text comprehension: Evidence from component skills. LANG COGNITIVE PROC 18(4): 443–468.

[8] Kirby JR, Savage RS (2008) Can the simple view deal with the complexities of reading? LITERACY 42(2): 75–82.

[9] Betjemann RS, Keenan JM, Olson RK, DeFries JC (2011) Choice of reading comprehension test influences the outcomes of genetic analyses. SCI STU READ 15(4): 363–382. 10.1080/10888438.2010.493965 PMC3143485 21804757

[10] White B, Kirby JR (2007) Depth of processing: Its relation to reading comprehension, component skills and cognitive processes. Poster presented at the annual conference of the Society for the Scientific Study of Reading, Prague, Czech Republic.

[11] Keenan JM, Betjemann RS, Wadsworth SJ, DeFries JC, Olson RK (2006) Genetic and environmental influences on reading and listening comprehension. J RES READ 29(1): 75–91.

[12] Harlaar N, Cutting L, Deater-Deckard K, DeThorne LS, Justice LM, et al (2010) Predicting individual differences in reading comprehension: A twin study. ANN DYSLEXIA 60: 265–288. 20814768 10.1007/s11881-010-0044-7 PMC2981603

[13] Byrne B, Samuelsson S, Wadsworth S, Hulslander J, Corley R, et al (2007) Longitudinal twin study of early literacy development: Preschool through Grade 1. READ WRIT 20: 77–102.

[14] Byrne B, Coventry WL, Olson RK, Samuelsson S, Corley R, et al (2009) Genetic and environmental influences on aspects of literacy and language in early childhood: Continuity and change from preschool to Grade 2. J NEUROLINGUIST 22: 219–236. 10.1016/j.jneuroling.2008.09.003 PMC2724015 20161176

[15] Olson RK, Keenan JM, Byrne B, Samuelsson S, Coventry WL, et al (2011) Genetic and environmental influences on vocabulary and reading development. SCI STU READ 15(1): 26–46. 10.1007/s11145-006-9018-x PMC3019615 21132077

[16] Curtis ME (1980) Development of components of reading skill. J EDUC PSYCHOL 72(5): 656–669.

[17] Hoover WA, Tunmer WE (1993) The components of reading. In: Thompson GB, Tunmer WE, Nicholson T, editors. Reading acquisition processes. Adelaide, South Australia: Multilingual Matters.

[18] Sticht T, James J (1984) Listening and reading. In: Pearson P, editor. Handbook of research on reading. New York: Longmans.

[19] Keenan JM, Olson RK, Betjemann RS (2009) Assessment and etiology of individual differences in reading comprehension. In: Wagner RK, Schatschneider C, Phythian-Sence C, editors. Beyond decoding: The behavioral and biological foundations of reading comprehension. New York: Guilford.

[20] Gernsbacher MA (1990) Language comprehension as structure building. Hillsdale, NJ: Erlbaum.

[21] Harlaar N, Hayiou-Thomas ME, Dale PS, Plomin R (2008) Why do preschool language abilities correlate with later reading? A twin study. J SPEECH LANG HEAR RES 51(3): 688–705. 18506044 10.1044/1092-4388(2008/049)

[22] Hayiou-Thomas ME, Harlaar N, Dale PS, Plomin R (2006) Genetic and environmental prediction from different aspects of preschool language and non-verbal ability to 7-year reading. J RES READ 29(1): 50–74.

[23] Keenan JM, Betjemann RS, Olson RK (2008) Reading comprehension tests vary in the skills they assess: Differential dependence on decoding and oral comprehension. SCI STU READ 12(3): 281–300.

[24] Hoosain R (1991) Psycholinguistic implications for linguistic relativity: A case study of Chinese. New Jersey: Lawrence Erlbaum Associates.

[25] Chow BWY, Ho CSH, Wong SWL, Waye M, Bishop DVM (2011) Genetic and environmental influences on Chinese language and reading abilities. PLoS ONE 6(2): e16640 doi: 10.1371/journal.pone.0016640 21347359 10.1371/journal.pone.0016640 PMC3037369

[26] Shu H, McBride-Chang C, Wu S, Liu H (2006) Understanding Chinese developmental dyslexia: Morphological awareness as a core cognitive construct. J EDUC PSYCHOL 98(1): 122–133.

[27] Yeung PS, Ho CSH, Chik PPM, Lo LY, Luan H, et al (2011) Reading and spelling Chinese among beginning readers: What skills make a difference? SCI STU READ 15(4): 285–313.

[28] Chik PPM, Ho CSH, Yeung PS, Wong YK, Chan D, et al (2012) Contribution of discourse and morphosyntax skills to reading comprehension in Chinese dyslexic and typically developing children. ANN DYSLEXIA 62: 1–18. 20835783 10.1007/s11881-010-0045-6 PMC3289786

[29] Yeung PS, Ho CSH, Chan D, Chung KK, Wong YK (under review) A Model of Reading Comprehension among Chinese Elementary School Children.

[30] Hoover WA, Gough PB (1990) The simple view of reading. READ WRIT 2: 127–160.

[31] Imaizumi Y (2003) A comparative study of zygotic twinning and triplet rates in eight countries, 1972–1999. J BIOSOC SCI 35(2): 287–302. 12664963 10.1017/s0021932003002876

[32] Chia KS, Lee JJM, Cheung P, Cheung KH, Seielstad M, et al (2004) Twin births in Singapore: A population-based study using the national birth registry. ANN ACAD MED SINGAP 33(2): 195–199. 15098633

[33] Rven JC, Court JH, Raven J (1995) Raven’s Coloured Progressive Matrices. UK: Oxford Psychologists Press.

[34] Dunn LM, Dunn DM (2007) Peabody Picture Vocabulary Test –4^th^ Edition. Minneapolis, MN: Pearson Assessments.

[35] Ho CSH, Chan DW, Tsang SM, Lee SH (2000) The Hong Kong Test of Specific Learning Difficulties in Reading and Writing. Hong Kong: Hong Kong Specific Learning Difficulties Research Team.

[36] Boker S, Neale M, Maes H, Wilde M, Spiegel M, et al (2011) OpenMx: An open source extended Structural Equation Modeling framework. PSYCHOMETRIKA 76(2): 306–317. 23258944 10.1007/s11336-010-9200-6 PMC3525063

